# Compliance with WHO IYCF Indicators and Dietary Intake Adequacy in a Sample of Malaysian Infants Aged 6–23 Months

**DOI:** 10.3390/nu8120778

**Published:** 2016-12-01

**Authors:** Geok Lin Khor, Sue Yee Tan, Kok Leong Tan, Pauline S. Chan, Maria Sofia V. Amarra

**Affiliations:** 1 Department of Nutrition and Dietetics, International Medical University, Kuala Lumpur 57000, Malaysia; sueyee_tan@imu.edu.my (S.Y.T.); kokleong_tan@imu.edu.my (K.L.T.); 2 International Life Sciences Institute South East Asia Region, Singapore 238959, Singapore; paulinechan@ilsisea.org.sg (P.S.C.); sofiaamarra@ilsisea.org.sg (M.S.V.A.)

**Keywords:** infant and young child feeding, dietary adequacy, core complementary feeding indicators, micronutrients, dietary diversity

## Abstract

Background: The 2010 World Health Organisation (WHO) Infant and Young Child Feeding (IYCF) indicators are useful for monitoring feeding practices. Methods: A total sample of 300 subjects aged 6 to 23 months was recruited from urban suburbs of Kuala Lumpur and Putrajaya. Compliance with each IYCF indicator was computed according to WHO recommendations. Dietary intake based on two-day weighed food records was obtained from a sub-group (*N* = 119) of the total sample. The mean adequacy ratio (MAR) value was computed as an overall measure of dietary intake adequacy. Contributions of core IYCF indicators to MAR were determined by multinomial logistic regression. Results: Generally, the subjects showed high compliance for (i) timely introduction of complementary foods at 6 to 8 months (97.9%); (ii) minimum meal frequency among non-breastfed children aged 6 to 23 months (95.2%); (iii) consumption of iron-rich foods at 6 to 23 months (92.3%); and minimum dietary diversity (78.0%). While relatively high proportions achieved the recommended intake levels for protein (87.4%) and iron (71.4%), lower proportions attained the recommendations for calcium (56.3%) and energy (56.3%). The intake of micronutrients was generally poor. The minimum dietary diversity had the greatest contribution to MAR (95% CI: 3.09, 39.87) (*p* = 0.000) among the core IYCF indicators. Conclusion: Malaysian urban infants and toddlers showed moderate to high compliance with WHO IYCF indicators. The robustness of the analytical approach in this study in quantifying contributions of IYCF indicators to MAR should be further investigated.

## 1. Introduction

Culture- and age-appropriate infant and young child feeding (IYCF) practices are well recognized as imperative for child health and survival. In 2008, the World Health Organization (WHO) recommended a set of population-level breastfeeding practices and food-related aspects of child feeding practices appropriate for children aged 6 to 23 months [1]. The questionnaire comprises eight core indicators and seven optional indicators of feeding practices, and offers the advantage of inter-country comparisons of IYCF practices.

In examining indicators of infant and young child feeding practices using data from Demographic and Health Surveys (DHS) of 46 countries between 2002 and 2008, Lutter et al. [2] reported that: (i) few infants and young children benefited from optimal complementary feeding practices; and (ii) less than one-third and only 21% of children aged 6 to 23 months met the minimum criteria for dietary diversity and received a minimum acceptable diet, respectively. Based on India’s National Family Health Survey (2005–2006), involving over 18,000 children aged zero to 23.9 months, Menon et al. [3] reported overall poor status of IYCF practices, especially indicators for complementary feeding, with 16% and 9% complying with minimum dietary diversity and minimum acceptable diet, respectively. A similar finding was reported by Marriot et al. [4], in assessing the WHO IYCF indicators of 14 low-income countries in Africa and Asia. The mean minimum dietary diversity adherence was found low, ranging from 11.3% for ages 6 to 11 months to 25.1% among those aged 18–23 months. The low prevalence of dietary diversity was reflected in poor compliance with minimum acceptable diet which was only 7.7% for infants 6 to 11 months of age and 16.3% for those aged 18–23 months. These recent reviews indicate room for improvement in young child feeding practices in low- to middle-income countries.

The International Life Sciences Institute of Southeast Asia region (ILSI SEA) Expert Panel on Infant and Young Child Nutrition, at its meeting in 2012, highlighted the importance for the region to undertake country-specific assessment of the IYCF indicators in association with measured dietary intakes and nutritional status of infants and young children [5]. In this respect, Cambodia, Indonesia, Philippines and Vietnam have periodic updates on IYCF practices, as reported by Demographic and Health Surveys (DHS) and/or Multiple Indicator Cluster Surveys (MICS) for these countries.

Malaysia is not covered by either the DHS or MICs, and relies instead on its National Health and Morbidity Surveys (NHMS) for IYCF data, such as the Third NHMS undertaken in 2006 [6] and the just-completed 2016 NHMS. There are concerns for inadequate feeding practices in Malaysia in view of recent findings of rather high prevalence of stunting and underweight among young children. Stunting among urban children nationwide was reported at 23.9% in ages 0.5–0.9 years [7], and underweight was reported at 19.6% among children aged 0–4 years [8]. Over-reliance on milk, leading to a lack of dietary diversity, was reported among Malaysian urban children aged one to three years [9]. Hence, this study was undertaken to assess the quality of young child feeding practices and dietary intake adequacy in a sample of infants and toddlers.

## 2. Methodology

This cross-sectional study was undertaken with the following objectives:

### 2.1. Objectives

1. To estimate the prevalence of compliance with the WHO IYCF indicators in a total sample of 300 infants and toddlers aged 6.0–23.9 months;
2. To determine dietary intake using a two-day weighed food records of a sub-group of infants and toddlers (*N* = 119), matched for age and sex with the total sample, with dietary intake adequacy computed as the mean adequacy ratio (MAR);
3. To determine the contributions of the WHO core complementary feeding indicators to the MAR values of the sub-group of subjects.

### 2.2. Subjects

The inclusion criteria for the selection of the study subjects were:

- Infants and children aged 6.0 to 23.9 months
- Malay ethnicity
- Families residing in the urban suburbs of Kuala Lumpur and Putrajaya
- Subjects attending licensed child care centers (registered with the Social Welfare Department of the Ministry of Women, Family and Community Development (Licensed child care centers are obliged to adhere to government regulations including caregiver to child ratio, standards of hygiene and menu guidelines.))
- Consent of the child care center management and parents/caregivers

The exclusion criteria were:

- Mentally or physically disabled infants and children
- Ill at the time of data collection
- Having dietary restrictions

The rationale for including only Malay infants and children of families from urban areas is to minimize the influence of cultural and socio-economic factors on feeding practices of infants and toddlers. Malay is the predominant ethnicity of the Malaysian population.

### 2.3. Total Sample Size for Determining Compliance with IYCF Indicators

The total sample size was estimated according to the guidelines of the WHO “Indicators for assessing infant and young child feeding practices [1] (Part 2 Measurements). Based on assuming an indicator estimate of 50% and a 95% confidence interval, a minimum number of 225 infants and young children aged 6.0–23.9 months was recommended. It was decided to have a total sample size of 300 subjects in case of dropouts and incomplete questionnaires. Data collection was carried out in 2013–2014.

A list of the licensed child care centers was compiled from various sources including the Association of Registered Care Providers of Selangor and the Ministry of Health Nutrition Division. The child care centers were contacted through emails and phone calls. Out of approximately 100 centers, about one-fifth gave permission for the conduct of this study. Using the snow-ball approach in going from one center to the next, approximately 100 infants and children who met the inclusion criteria were recruited for each of these age groups: 6.0–11.9 months, 12.0–17.9 months and 18.0–23.9 months, with almost an equal proportion of males and females in each age group. As the participants were recruited by convenience sampling, they are not deemed as representative of urban Malay toddlers.

The WHO IYCF indicators questionnaire was translated to the Malay language and pretested before use on five Malay mothers. This is to improve the language, technical contents and length of time used for the interview. Calculation for compliance with each IYCF indicator was in accordance with the formula recommended by WHO [1] (Part 2 Measurements).

### 2.4. Sub-Group of Toddlers for Two-Day Weighed Food Records and Compliance with Core Complementary Feeding

Only a sub-group of subjects was included for the two-day weighed food records part of the study, given that weighing of food intake was time-consuming. The sub-group of subjects was matched for age and sex to the total sample. In this way, the sub-sample comprised approximately 40 subjects in each of the three age groups of 6.0–11.9 months, 12.0–17.9 months and 18.0–23.9 months, making a total of 120 subjects. This age disaggregation is in accordance with the recommendation for studying the core complementary feeding indicators [1]. Each of the age groups consisted of approximately an equal proportion of male and female subjects. At the end, 119 subjects were included as one questionnaire was found to be incomplete. 

Trained research assistants with at least a bachelor-level qualification in nutrition or dietetics carried out the weighing of food intake for two days when the child was in the center from about 7 a.m. until 5 p.m., Monday to Friday. All foods consumed were weighed for two consecutive weekdays but not on a weekend, as the day care centers are closed during the weekends.

For solid or semi-solid foods, the research assistants prepared a duplicate plate of the food items that were given to the child. The individual items were weighed using a kitchen scale that has a weight capacity of 2 kg and a weight graduation of 1 gm (TANITA-KD160WH). The amounts not consumed by the child were deducted from the total amounts weighed. The research assistants checked for ingredients used in preparing the food items by asking the center manager or person who prepared the foods or beverages.

For recording food and beverages consumed by children who were fed individually by the center helpers, the research assistants first identified the ingredients used in preparing the food or beverage, and then estimated the amounts of the food/beverage consumed by the child, based on the serving spoon or cup. As the entire procedure of observation, weighing and calculation for each child was time-consuming, a research assistant could complete the procedure for only three to four subjects a day, on average.

Parents/caregivers were instructed to record foods and beverages, in terms of description and quantity, taken by their child from the time he/she was picked up from the child care center until the following morning when the child was brought back to the center. The research assistants checked the forms and sought clarifications from parents/care givers, particularly for the quantities and types of constituents in the food or beverage consumed at home or outside.

## 3. Statistical Analyses

Compliance with the IYCF indicators was reported in percentage. Dietary intake was computed in terms of percentage of meeting the Malaysian dietary recommendations (Recommended Nutrient Intakes (RNIs) [10]. The mean adequacy ratio (MAR) was used as an overall measure of dietary adequacy. Several studies have reported MAR values to be positively associated with other indexes of dietary quality [4,11]. In order to compute MAR, one has to calculate first the nutrient adequacy ratio (NAR), which is defined as the ratio of a subject’s nutrient intake to the recommended nutrient intake (RNI) of the assessed nutrient, appropriate for the age and sex of the subject.

NAR = Actual nutrient intake (per day)/Recommended nutrient intake (RNI)
(1)

The value for MAR is calculated as the total of all the NARs divided by the total expressed as a percentage. MAR has a range from 0% to 100% with 100% as the ideal [12].

MAR = ∑ NARs/Total number of nutrients
(2)

For computing MAR, each of the NARs was truncated as 1, so that a high intake of one nutrient could not compensate for the low intake of another nutrient, as originally conceived by Malden et al. [13] and cited by Vieux et al. [14].

Multinomial logistic regression was used to assess the odds of compliance with the core complementary feeding indicators as the nominal independent variable, and MAR values as the dependent variable. All analyses were performed with SPSS version 22.0 (SPSS Inc., Chicago, IL, USA). A level of <0.05 was set for statistical significance.

### Ethics Approval and Consent

The study proposal was approved by the Joint Committee on Research and Ethics of the International Medical University, Kuala Lumpur, Malaysia on 23 September 2013. (Project ID No. IMU R 123/2013). The Study Information Sheet and Written Consent Form in the Malay and English language were also approved by the IMU ethics committee. The Study Information Sheet comprised the following information: What is the purpose of this research? Why you are invited to this research? What is involved in this research? Is there any danger? How does this research help me? Contact persons for further information. After we had explained the information to the parents, one of them signed the Written Consent Form allowing their child to participate in the study.

## 4. Results

### 4.1. Total Sample Compliance with IYCF Indicators (N = 300)

The total sample size of 300 children consisted of 105 subjects aged 6.0–11.9 months, 87 aged 12.0–17.9 months, and 98 aged 18.0–23.9 months, with about an equal proportion of male and female subjects in each age group. These children were from young families with mothers having an average age of 30.9 ± 0.3 years, and with 1.4 ± 0.1 of children aged three years or less per family, on average. 

Out of the WHO set of 15 IYCF indicators, data was collected for 12 indicators only, as the other three indicators referred to feeding practices for infants aged from zero to under 6 months, whereas these study subjects were aged 6.0–23.9 months. The subjects showed high compliance for several of the assessed indicators, namely (i) Children ever breastfed (99.3%); (ii) Timely introduction of complementary foods at 6–8 months (97.9%); (iii) Minimum meal frequency among non-breastfed children aged 6–23 months (95.2%); and (iv) Consumption of iron-rich foods at 6–23 months (92.3%) (Table 1). In contrast, the other indicators were achieved by relatively lower proportions of the subjects, and these include indicators related to breastfeeding beyond 6 months of age: (i) Continued breastfeeding at 2 years (38.3%); (ii) Continued breastfeeding at 1 year (57.1%); and (iii) Age-appropriate breastfeeding among children aged 6–23 months receiving breast milk as well as solids in the previous day (55.9%).

The minimum acceptable diet indicator was also found to have relatively low compliance among the non-breastfed children (39.5%) and breastfed children (50.6%). The minimum acceptable diet indicator combines compliance with the minimum dietary diversity and minimum meal frequency indicators. Actually, the subjects showed quite high compliance with meeting the latter two indicators separately, which is 78% for minimum dietary diversity, and 95.2% and 69.3% for minimum meal frequency among non-breastfed and breastfed children, respectively. However, in computing the minimum acceptable diet indicator for non-breastfed subjects, the formula includes the criteria of receiving two milk feeds, in addition to meeting the criteria for dietary diversity and meal frequency; moreover, the dairy group is excluded when calculating dietary diversity to avoid “double counting” [1] (WHO Part 2 Measurements). Consequently, the acceptable diet formula becomes more stringent to attain, resulting in a relatively lower prevalence of compliance among the non-breastfed.

### 4.2. Compliance with Core Complementary Feeding Indicators by Sub-Group (N = 119)

Compliance with four of the five core indicators for complementary feeding was investigated among the sub-group of subjects, as only a small number (*N* = 13) of subjects (aged 6 to 8 months) was eligible for computing the indicator on “Timely introduction of solids“, which was hence excluded. The sub-group showed generally high compliance with the core complementary feeding indicators (Table 2). There was high compliance with (i) Timely introduction of solid, semi-solid or soft foods (92.3%); (ii) Consumption of iron-rich foods (92.4%); (iii) Minimum meal frequency among both breastfed and non-breastfed children aged 6–23 months (91.7% and 85.5%, respectively); as well as (iv) Minimum dietary diversity (83.2%). A low prevalence for the minimum acceptable diet indicator for non-breastfed (34.6%) and breastfed (68.8%) infants was recorded for the sub-group.

### 4.3. Dietary Intake Adequacy of Sub-Group (N = 119)

Based on the two-day weighed food intake, dietary intake adequacy was computed and described as meeting the Malaysian recommendations for energy and nutrient intake (RNI) [10]. The nutrient adequacy ratio (NAR) value exceeded 1.0 for energy, protein, calcium and iron, indicating that their intakes achieved more than 100% of the recommended levels (RNIs) (Table 3). Mean iron and protein intake were double the RNI recommendation levels, while those for energy and calcium exceeded 100%. The likely dietary sources for these nutrients were animal food including milk, chicken meat, fish and eggs. As for the NAR values for the rest of the nutrients, they ranged from 0.50 for vitamin A to 0.84 for zinc, denoting intake of these nutrients met 50% to 84% of the RNIs, respectively. Relatively high proportions of the subjects achieved the RNI for protein (87.4%) and iron (71.4%), while just over half managed to do so for calcium (56.3%) and energy (56.3%). The proportions of the subjects meeting the rest of the micronutrients were low and these include vitamin A, thiamin, vitamin C niacin, riboflavin and zinc. This finding suggests that the subjects were not consuming sufficient vegetables, fruits, legumes and whole grains.

For this study, it was arbitrarily decided to compute NAR for energy and nutrients taken by the subjects that met at least 40% of the Malaysian RNIs. Hence, NAR was determined for energy and the following nine nutrients: protein, calcium, iron, zinc, vitamin A, vitamin C, thiamin, riboflavin and niacin. The value for MAR was then calculated as ∑ NARs divided by the total (10 in this case) expressed as a percentage. 

Overall, the mean adequacy ratio (MAR) value computed was 0.67, indicating that the dietary intake of the subjects met, on average, 67% of the combined RNIs for energy and the nine nutrients under consideration in this study.

### 4.4. Contributions of Core Complementary Feeding Indicators to Dietary Intake Adequacy (N = 119)

Multinomial logistic regression was used to determine the contributions of the four WHO core complementary feeding indicators as the independent variables to dietary intake adequacy based on MAR values as the dependent variables. Since MAR = 0.67 was generated from the weighed dietary intake in this study, MAR > 0.6 was selected for comparison with MAR ≤ 0.5. Among the core complementary feeding indicators, minimum dietary diversity contributed highest to dietary intake adequacy (OR 11.10; 95% CI: 3.09, 39.87) (*p* = 0.000) (Table 4). The other core complementary feeding indicators showed lower odds of contributing to intake adequacy for the same MAR values. The minimum dietary diversity indicator also showed the highest odds of leading to dietary intake adequacy (OR 5.49; 95% CI: 1.35, 22.22) (*p* = 0.017) with MAR set at a lower level of (0.5 < MAR ≤ 0.6) that is, overall dietary adequacy between 50.1% and 60% (Table 4). Hence, these results suggest that, for this sample of Malaysian urban toddlers, compliance with minimum dietary diversity is of key importance towards attaining dietary intake adequacy. 

## 5. Discussion

In a sample of urban Malaysian infants and toddlers aged 6 to 23 months, this study highlighted (i) moderate rates of continued breastfeeding beyond 6 months of age; (ii) high proportions of compliance with timely introduction of complementary foods and receiving iron-rich foods; and (iii) moderate to low proportions met the acceptable diet indicator (minimum dietary diversity and minimum meal frequency). Achieving the indicator on acceptable diet is important as it reflects compliance with both the qualitative and quantitative aspects of complementary feeding. Studies have well documented the significant relationships between attaining a minimum acceptable diet and child nutritional status. In assessing the WHO IYCF indicators of 14 low-income countries in Africa and Asia, Marriot et al. [4] found children consuming a minimum acceptable diet had a significantly lower overall probability of being underweight and stunted. Based on India’s National Family Health Survey (2005–2006), involving over 18,000 children aged zero to 23.9 months, Menon et al. [3] showed the acceptable diet indicator as a strong predictor of stunting and underweight.

Dietary intake adequacy in terms of MARs was shown to be influenced most by the minimum dietary diversity indicator. This finding is in line with growing global evidence of the usefulness of dietary diversity in predicting dietary quality, including micronutrient intake adequacy among infants and young children [12,15,16,17]. In evaluating cross-country patterns based on DHS data, Jones et al. [18] reported that achieving minimum dietary diversity reduced the odds of stunting among children in India, Bangladesh and Zambia. Data from the 2007 Bangladesh Demographic and Health Survey of 1508 children aged 6 to 23 months also showed that those who achieved minimum dietary diversity had a significantly higher height for their age [19]. Nguyen et al. [20] reported a strong positive association between maternal and child dietary diversity in Bangladesh, Vietnam and Ethiopia, and hence advocated the measurement of both maternal and child dietary diversity. A low dietary diversity score was significantly associated with poor nutritional status of indigenous children in Malaysia [21]. A lack of dietary diversity is often reported in low-income populations as their diets are predominantly based on starchy staples, as animal food sources are costly and may not be readily available.

Countries are encouraged to conduct periodic surveys using the WHO IYCF indicators to enable comparing trends of breastfeeding and complementary feeding practices within countries and among countries, as well as for identifying populations at risk and evaluating the impacts of interventions [18]. Countries in Southeast Asia generally showed higher compliance with timely introduction of complementary foods than that for dietary diversity, meal frequency and acceptable diet (Table 5). These findings suggest that complementary foods are provided in a timely manner, but the diets tend to lack variety and frequency as recommended. Hence, owing to low achievements of dietary diversity and meal frequency, compliance with a minimum acceptable diet is generally low in the region. 

### Limitations of Study

The relatively small sample size and the homogeneous subjects preclude generalizing the results to other socio-economic and ethnic population groups of Malaysia. As a cross-section study, interpretation of causality of the data is restricted. The accuracy of the results is also limited by the methods of data collection, particularly relying on weighing of foods and food intake recording by parents/caregivers. There may be bias in the selection of subjects using the convenient snow-ball approach. The dietary results obtained should not be extended to children who do not attend licensed day care centers where the menus are based on the recommendations of the Ministry of Health. 

## 6. Conclusions

Continued efforts remain essential for improving feeding practices to ensure adequate and quality complementary feeding in Malaysia and the region. This study provides an analytic approach at examining the contributions of core complementary feeding indicators to dietary intake adequacy. This approach should be validated with larger subject samples from different socio-economic statuses to verify its usefulness at different population levels.

## Figures and Tables

**Table 1:** Compliance (%) with WHO IYCF indicators [1] (WHO, 2010) by total sample (*N* = 300).

**Core Indicators**		% (*N*) *
1. Early initiation of breastfeeding		
	Proportion of children born in the last 24 months who were put to the breast within one hour of birth	76.3 (300)
2. Exclusive breastfeeding under 6 months		-
3. Continued breastfeeding at 1 year		
	Proportion of children aged 12–15 months who are fed breast milk during the previous day	57.1 (56)
4. Timely introduction of solid, semi-solid or soft foods		
	Proportion of infants aged 6–8 months who receive solid, semi-solid or soft foods during the previous day	97.9 (47)
5. Minimum dietary diversity		
	Proportion of children aged 6–23 months who received foods from 4 or more food groups during the previous day	78.0 (300)
6. Minimum meal frequency		
	Breastfed children: Proportion of breastfed children aged 6–23 months who received solid, semi-solid, or soft foods the minimum number of times or more during the previous day	69.3 (163)
	Non-breastfed children: Proportion of children aged 6–23 months who received solid, semi-solid or soft foods or milk feeds the minimum number of times or more during the previous day	95.2 (125)
	Total:	80.6 (288)
7. Minimum acceptable diet		
	Breastfed children: Proportion of children aged 6–23 months who had at least a minimum dietary diversity and the minimum meal frequency during the previous day	50.6 (166)
	Non-breastfed children: Proportion of children aged 6–23 months who received at least 2 milk feedings and had at least the minimum dietary diversity and the minimum meal frequency during the previous day	39.5 (129)
	Total:	45.8 (295)
8. Consumption of iron-rich or iron-fortified foods		
	Proportion of children aged 6–23 months who receive an iron-rich food or iron-fortified food that is specially designed for infants and young children, or that is fortified in the home during the previous day	92.3 (298)
**Optional Indicators**		
9. Children ever breastfed		
	Proportion of children born in the last 24 months who were ever breastfed	99.3 (300)
10. Continued breastfeeding at 2 years		
	Proportion of children aged 20–23 months who received breast milk during the previous day	38.3 (47)
11. Age-appropriate breastfeeding		-
	Proportion of children aged 6–23 months who received breast milk, as well as solid, semi-solid or soft foods during the previous day	55.9 (161)
12. Predominant breastfeeding under 6 months (0–5.9 months)		-
13. Duration of breastfeeding (0–5.9 months)		-
14. Bottle feeding		
	Proportion of children aged 0–23 months who are fed with a bottle during the previous day	80.4 (296) **
15. Milk feeding frequency for non-breastfed children		
	Proportion of non-breastfed children 6–23 months of age who received at least 2 milk feedings during the previous day	91.3 (127)

* Number of eligible subjects; ** Age studied 6.0–23.9 months.

**Table 2:** Compliance (%) with WHO core complementary feeding indicators by sub-group (*N* = 119).

* Core Complementary Feeding Indicators	Compliance% (*N*)
Minimum dietary diversity	83.2 (119)
Minimum meal frequency	
Breastfed	85.5 (62)
Non-breastfed	91.7 (48)
Minimum acceptable diet	
Breastfed	68.8 (64)
Non-breastfed	34.6 (52)
Consumption of iron-rich or iron-fortified foods	92.4 (119)

* Timely introduction of solid, semi-solid or soft foods indicator was excluded owing to the small number of subjects who were eligible (*N* = 13 aged 6 to 8 months).

**Table 3:** Energy and nutrient intake adequacy (*N* = 119).

Dietary Intake ^†^	Percentage of the Recommended Nutrient Intake (RNI) Achieved % ± SD	^a^ Nutrient Adequacy Ratio (NAR) Value Mean ± SE
energy	110.7 ± 49.2	1.14 ± 0.05
protein	202.8 ± 101.9	2.07 ± 0.10
calcium	127.5 ± 92.6	1.32 ± 0.10
iron ^b^	216.7 ± 181.2	2.44 ± 0.21
zinc	75.7 ± 70.2	0.84 ± 0.10
thiamin	47.6 ± 34.6	0.55 ± 0.06
riboflavin	76.0 ± 51.5	0.82 ± 0.06
niacin	66.5 ± 53.5	0.77 ± 0.07
vitamin C	41.5 ± 39.7	0.57 ± 0.07
vitamin A	42.2 ± 39.6	0.50 ± 0.06
^c^ Mean adequacy ratio (MAR)		0.67 ± 0.02

**^†^** based on two-day weighed food record; ^a^ NAR for a given nutrient is the ratio of a subject’s nutrient intake to the recommended nutrient intake of the assessed nutrient, appropriate for the age and sex of the subject; ^b^ based on 15% bioavailability; ^c^ Mean adequacy ratio (MAR) was calculated as the total of all the NARs (∑ NAR for energy and nine nutrients) divided by the total, expressed as a percentage.

**Table 4:** Contributions of WHO core complementary feeding indicators to dietary intake adequacy (*N* = 119) **^†^**.

Dietary Intake Adequacy Based on Mean Adequacy Ratio (MAR)	Minimum Dietary Diversity	Minimum Meal Frequency	Minimum Acceptable Diet	Consumption of Iron-Rich/Fortified Food
	Odds Ratio (95% CI)			
^b^ MAR > 0.6 (more than 60.0%)	11.10 (3.09, 39.87), *p* = 0.000	6.70 (1.44, 31.16), *p* = 0.015	4.03 (1.17, 13.87), *p* = 0.027	2.73 (0.45, 16.48), *p* = 0.273
0.5 < ^a^ MAR ≤ 0.6 (50.1%–60.0%)	5.49 (1.35, 22.22), *p* = 0.017	1.60 (0.35, 7.30), *p* = 0.544	2.96 (0.75, 11.67), *p* = 0.121	1.33 (0.20, 9.00), *p* = 0.768

Above results generated using multinomial logistic regression whereby the reference category is MAR ≤ 0.5; **^†^** Dietary adequacy defined as meeting the combined RNIs for energy and nine nutrients (protein, calcium, iron, zinc, vitamin A, vitamin C, thiamin, riboflavin and niacin); NAR = 10 Nutrient adequacy ratio (NAR) for a given nutrient is the ratio of a subject’s intake to the current recommended nutrient intake of the assessed nutrient, appropriate for the age and sex of the subject. Mean adequacy ratio (MAR) is the total of all the NARs (∑ NAR for energy and nine nutrients) divided by the 10, expressed as a percentage; 0.5 < ^a^ MAR ≤ 0.6: meeting between 50.1% to 60.0% of the combined RNIs for the 10 NARs; ^b^ MAR > 0.6: meeting more than 60% of the combined RNIs for the 10 NARs.

**Table 5:** Comparison of WHO IYCF practices among countries in Southeast Asia *.

Core Complementary Feeding Indicators	Cambodia 2014 DHS	Indonesia 2012 DHS	Philippines 2008 DHS	Vietnam 2013–2014 MICS5	** Malaysia 2013–2014 Present Study
Timely introduction of complementary foods	82	91.0	89	90.7	97.9
Minimum dietary diversity	48.0	58.2	78.7	76.9	78.0
Minimum meal frequency					
breastfed					
Non-breastfed	74.0	61.4	80.7		69.3
Total	68.0	78.7	48.2	90.5	95.2
Minimum acceptable diet					
breastfed	32.0	34.2	68.2	62.4	50.6
Non-breastfed	26.0	43.0	40.5	54.5	39.5
Consumption of iron-rich/iron-fortified foods	75.8	67.5	78.3		92.3

* Cambodia, Indonesia, Philippines and Vietnam covered by Demographic and Health Surveys (DHS) or Multiple Indicators Cluster Surveys (MICS) [22,23,24,25]; ** *N* = 300.
